# Cross-strait parasitological research priorities arrived at by historical tracking and advanced dialogue

**DOI:** 10.1186/2049-9957-3-40

**Published:** 2014-12-01

**Authors:** Jyh-Wei Shin, Jia-Xu Chen, Dong-Hui Zhang, Wei-Chen Lin, Bo Shen, Min-Jun Ji

**Affiliations:** Department of Parasitology, National Cheng Kung University, Tainan, Taiwan; The National Institute of Parasitic Diseases, Chinese Center for Disease Control and Prevention, Shanghai, China; Department of Pathogen Biology, Nanjing Medical University, Nanjing, Jiangsu China

**Keywords:** Cross-strait meeting, Parasitological research priorities, Epidemiology

## Abstract

To further enhance dialogue and promote cross-strait cooperation in the prevention and control of parasitic diseases, this paper reviewed the progress and current challenges in the cross-strait control and research of parasitic infections, based on three cross-strait meetings on parasitological research in the last decade. The major outcome of the 3rd Meeting of Cross-Strait Parasitological Research held in April 2013 was identifying the research priorities for parasitological research.

**Electronic supplementary material:**

The online version of this article (doi:10.1186/2049-9957-3-40) contains supplementary material, which is available to authorized users.

## Multilingual abstracts

Please see Additional file 1 for translations of the abstract into the six official working languages of the United Nations.

## Review

The main purpose of this paper is to enhance dialogue and promote cross-strait cooperation in the prevention and control of parasitic diseases. This paper put forward some research priorities based on the analysis of epidemic situation of parasitic diseases on both sides of the Strait.

## Background

Sir Patrick Manson travelled to Formosa, now Taiwan, as a medical officer to the Chinese Imperial Maritime Customs in 1866, where he started a lifelong career in the research of tropical medicine for diseases such as paragonimiasis, elephantiasis and malaria. After five years in Takao (now Kaohsiung), Taiwan, he transferred to Amoy (now Xiamen, mainland China) on the Chinese coast where he worked for another 13 years. Then, he went to Hong Kong to open the first medical school there. To honour Sir Manson’s contributions to tropical medicine, the 1st Meeting of Cross-Strait Parasitological Research was held from September 12 to 14, 2003, at the National Yang-Ming University, in Taipei, Taiwan.

After eight years, Dr. Xiao-Nong Zhou and Dr. Jyh-Wei Shin cooperatively coordinated the 2nd Meeting of Cross-Strait Parasitological Research, which was held from August 31 to September 1, 2011, at the National Cheng Kung University, in Tainan. There were five principal sessions in the meeting: food-borne, vector-borne, soil-transmitted, zoonotic and opportunistic parasitic diseases.

Five objectives were agreed upon by all partners in the second cross-strait meeting: 1) to continue the cooperative mechanism that was an outcome of the 1st Meeting of Cross-Strait Parasitological Research; 2) to understand the historical and current epidemiology of cross-strait parasitic infections; 3) to share new technology in cross-strait parasitological research; 4) to create a cross-strait platform for young scientists and 5) to establish a preventive channel for the cross-strait control of imported parasitic infections.

In order to achieve these objectives, the 3rd Meeting of Cross-Strait Parasitological Research was held from April 17 to 18, 2013, in Nanjing. In the meeting, five principal sessions were focused on: 1) food-borne parasitic diseases; 2) opportunistic parasitic diseases; 3) vector-borne parasitic diseases; 4) new technology for diagnostics of parasitic diseases, and 5) prevention of global health and parasitic diseases.

This paper reviews the outcomes of the third cross-strait meeting and identifies the future research priorities for parasitological research.

## Epidemiology of cross-strait parasitic infections

After undergoing long-term mass prevention and control, some important parasitic diseases have been well controlled, and even eliminated, in Taiwan and mainland China. However, due to their different geographical characteristics and economic conditions, Taiwan and mainland China encounter different disease spectrums that require different control strategies. In mainland China, especially, there is still a considerable burden of parasitic diseases. The current status of some of the important parasitic diseases are discussed below and the relative data is shown in Table 1.

**Table 1:** **Current status of some of the major parasitic diseases in mainland China and Taiwan**

	*Mainland China*	*Taiwan*
Malaria	A total of 2,718 cases including 2,474 imported cases (901 *Pv* and 1,403 *Pf*) in 2012	Successful eradication since 1965
Leishmaniasis	A mean of about 400 cases per year with 97.71% of cases concentrated in the Xinjiang, Gansu and Sichuan provinces	NI
Schistosomiasis	13 acute cases in 2012 and approximately 68 million individuals at risk	*Schistosoma japonicum* Taiwan Changhua strain infects some mammal animals and rarely affects humans
Filariasis	The elimination of filariasis confirmed by the WHO in 2005	Eradication of bancroftian filariasis in Kinmen since 1978
Intestinal helminthiasis	Well-controlled intestinal helminthiasis, with 6.9 million infected people in 2012	Rare (about 0.4% infection rate) in most regions in Taiwan except for the mountain regions
Clonorchiasis	15 million people infected with clonorchiasis	NI
Cysticercosis	7 million people infected with cysticercosis and 550,000 people infected with taeniasis	NI
Echinococcosis	380,000 cases of echinococcosis in 7 western provinces	NI
Trichinellosis	20 million people infected with trichinellosis	NI
Others	400 angiostrongyliasis cases	NI

NI: no information.

### Parasitic infections in Taiwan

Malaria was highly endemic in Taiwan before World War II (1939–1945), with an estimated 1.2 million cases out of a population of six million at the time [1]. The Malaria Eradication Program in Taiwan was successfully carried out between 1947 and 1965 through a combined effort of the government, the World Health Organization (WHO), the United Nations International Children’s Emergency Fund (UNICEF) and The United States Agency for International Development (USAID), and involving the participation of the communities. The last indigenous case of *Plasmodium falciparum* occurred on July 22, 1961, of *P. vivax* on December 24, 1961 and of *P. malariae* on November 11, 1962. The successful eradication of malaria in Taiwan was certified by a three-member WHO evaluation team in December 1964. In 1965, Taiwan was registered by the WHO on its list of countries where malaria eradication had been achieved [2]. The subsequent 40 years were successfully maintained as malaria-free. In the last decade – except for a few scattered recurrent cases in 1995 of induced infection cases at the Veterans General Hospital in Taipei – there have been an average of 38 imported cases per year but none have been indigenous.

Filariasis was once an important parasitic disease in Taiwan. During field observations conducted from June 1958 to August 1960, it was assumed that filariasis was introduced into the southwest part of Taiwan proper from the coastal areas of mainland China, where filariasis (especially due to *Wuchereria bancrofti*) was endemic. The disease was thought to come via the Pescadores Islands (now Penghu) as, historically, the natives of Taiwan originated from the Fujian and Guangdong provinces, first settling in Penghu and later reaching the southwest coast of Taiwan. A control programme for bancroftian filariasis was conducted in Kinmen proper from 1970 to 1982. The combined method of mass chemotherapy with diethylcarbamazine (DEC) for microfilarial carriers and larvicide with sumithion for mosquitoes was used. In 1978, bancroftian filariasis was eradicated on the Kinmen islands [3, 4].

In Taiwan, a parasite control plan targeting children in primary schools began in 1971, and reduced the prevalence of parasitic diseases from 73% to 0.19% in 1985. Between 1976 and 1985, the prevalence of *Ascaris lumbricoides* and *Trichuris trichiura* decreased from 6.8% and 11.4% to 0.3% and 0.4%, respectively [5]. Although, the control plan only involved primary school-age children, which was the major infected group, this still led to the overall decrease in the prevalence rate. *Enterobius vermicularis* is another important nematode which mainly infected preschool and under 10-year old children in Taiwan. The infection rates were estimated at a mass screening in the first year after the children started school, and the prevalence decreased from 16.3% in 1991 to 0.6% in 1996 [6]. The latest mass screening of the pinworm infection among children attending preschools in Taipei City was held in 2005. In this study, the prevalence was 0.40% (197/49,541), which remained unchanged compared to what was previously reported [7]. In conclusion, intestinal helminthiasis is rare in most regions in Taiwan except in the mountainous regions. We propose that regular pinworm screening and treatment programmes continue in some parts of Taiwan.

### Parasitic infections in mainland China

The unsatisfactory public health situation during the 1950s and 1960s caused 70 million people to suffer from malaria, schistosomiasis and filariasis [8]. In 1956, the State Council adopted a policy to fight malaria, leishmaniasis, schistosomiasis, filariasis and hookworm disease, and a number of important parasitic diseases have been controlled or eradicated in the past 60 years.

Malaria, an ancient disease, has been the leading infectious disease threat in most areas of mainland China. It is estimated that there were about 30 million cases of malaria before 1949. Because of the Chinese government’s consistent efforts to control and prevent malaria, the number of total malaria cases reported has dramatically declined from 24 million in the 1970s to 14,491 cases in 2009. To fulfil the commitments of the Global Malaria Eradication Programme advocated by the UN’s summit on the Millennium Development Goals, the Chinese government launched the National Action Plan for Malaria Elimination in 2010 to continue until 2020. In 2012, 2,718 malaria cases – including 1,080 *P. vivax* malaria cases, 1,419 *P. falciparum* malaria cases, 44 mixed infection cases of *P. vivax* and *P. falciparum*, 56 cases of *P. ovale* and *P. malariae*, and 119 unidentified cases, with 15 deaths – were reported via the infectious diseases reporting system from 620 counties of the 31 provinces in mainland China. These data showed that imported malaria cases dominate, accounting for 91.0% of the total malaria cases in 2012 [9]. Thus, the control and prevention of imported malaria should be one of the emphasis for the elimination of malaria.

Visceral leishmaniasis (also called ‘kala-azar’) caused by *Leishmania donovani* was once prevalent in 17 provinces, municipalities and autonomous regions, especially in the rural areas north of the Yangtze River. As a result of effective control measures, including powerful drug treatments with pentavalent antimony, the eradication of sandflies and the culling of infected dogs, the disease was almost eliminated in China between 1958 and 1960 [10], with the number of cases falling from 530,000 in 1951 to 10,000 in 1959. Thereafter, there were only a few sporadic cases in six provinces or autonomous regions in western China, namely Xinjiang, Gansu, Sichuan, Shaanxi, Shanxi and Inner Mongolia [11]. In recent years, endemic regions spread, the prevalence increased and even an outbreak occurred in China due to global warming and population movement [12]. Between 2005 and 2010, a total of 2,450 cases of visceral leishmaniasis were reported in China through the web-based National Diseases Reporting System (NDRS), of which 97.71% were concentrated in the Xinjiang autonomous region, and the Gansu and Sichuan provinces. Infants and young children are more likely to suffer from leishmaniasis.

Schistosomiasis japonica caused by the *Schistosoma japonicum* Chinese mainland strain was epidemic throughout 12 provinces in the 1950s and posed a significant public health problem. Around 100 million people were at risk of infection, 11.6 million people were infected, 1.2 million cattle were infected and the habitat area of the intermediate host snail *Oncomelania hupensis* was 14.3 billion m^2^. Over the past 60 years, the Chinese government has adopted a series of policies and measures to control schistosomiasis. The time course can be divided into three phases: disease elimination strategy through snail control (1950s until early 1980s), the morbidity control strategy based on chemotherapy (mid-1980s to 2003) and the integrated control strategy (from 2004 onwards) [13]. By 1995, five provinces, municipalities and autonomous regions, including Guangdong, Shanghai, Fujian, Guangxi and Zhejiang, had blocked the transmission of schistosomiasis japonica [14], whilst the Sichuan, Yunnan and Jiangsu provinces reached the criteria of transmission control (both human and livestock prevalence less than 1%) in 2008, 2009 and 2011, respectively. Four other provinces, namely Hubei, Hunan, Jiangxi and Anhui, reached the criteria of infection control (both human and livestock prevalence less than 5%) in 2008. One of the most important goals is for the prevalence rate in humans to reduce to less than 1% in all counties by 2015. To achieve this target, a comprehensive integrated approach focused on the elimination of infection sources in major endemic regions is being implemented. The number of infected cases has reduced by over 97% since the 1950s, reaching the lowest historic level of 240,597 infections in 2012 [15]. Moreover, acute infections were also significantly reduced. From 2004 to 2012, the number of acute cases dramatically declined from 816 to 13. The habitat area of *O. hupensis* was estimated to be 3.69 billion m^2^, which is about 25% less than in 1950s. However, we should note that there are still approximately 68 million individuals at risk of infection.

Lymphatic filariasis is caused by *Wuchereria bancrofti* and *Brugia malayi*, which reside in the body’s lymph nodes and result in the swelling and deformed growth of limbs and genitals. Sixty years ago, there were 31 million cases of lymphatic filariasis and 330 million people were at risk of infection in mainland China. Given that DEC was safe and effective for treating microfilaremia, mass treatment of the whole population was undertaken and all inhabitants above five years of age with or without microfilaremia received DEC and even ate DEC-fortified table salt in order to drastically eliminate infection sources. By 1994, all 864 endemic counties/cities in 15 provinces had achieved the criteria for effective control of filariasis and the microfilaria rate was less than 1% [16]. In 2005, the WHO verified that China became the first developing country in the world to eliminate filariasis. China continues its surveillance of the disease throughout the country and is strengthening its methods to prevent imported filariasis cases.

With the development of the economy and the improvement in people’s health habits, the prevalence of soil-transmitted nematodes had also markedly declined. Comparing 2003 with 1990, the prevalence of hookworms, *Ascaris* spp. and *Trichuris* spp. infections reduced by 60.7%, 71.3% and 73.6%, respectively. The number of people infected with soil-transmitted nematodes decreased from 536 million in 1990 to 129 million in 2003, of which 39.3, 85.9 and 29.1 million represented infections with hookworms, *A. lumbricoides* and *T. trichiura*, respectively [17, 18]. The infection rates of soil-transmitted nematodes in populations continue to decline, and the number of infected people decreased from 20.9 million in 2006 to 6.9 million in 2012. However, because egg pollution rates in soil were higher than the infection rates of the population, the risk of transmission still exists.

Although the above parasitic diseases has been effectively controlled, food-borne parasitic diseases are taken more seriously due to the great impact placed on food safety and public health. Food-borne diseases include clonorchiasis, cysticercosis and taeniasis, echinococcosis, trichinellosis and angiostrongyliasis [19]. *Clonorchis sinensis* induces an inflammatory reaction in the bile ducts and sometimes leads to periductal fibrosis and cholangiocarcinoma [20]. The prevalence of clonorchiasis increased from 0.37% between 1988 and 1992 to 0.58% between 2001 and 2004. Meanwhile, another survey reported that the prevalence was 2.40%. At present, the infected population stands at 15 million and most cases are concentrated in the Guangdong province and the Guangxi autonomous region [21]. Human cysticercosis caused by the larval stage of *Taenia solium* occurred in 29 provinces, municipalities and autonomous regions, and seven million people are infected. In the Yunnan, Guizhou and Sichuan provinces, several minorities prefer to eat raw pork and, hence, are at a higher risk of developing cysticercosis. Human taeniasis is caused by the tapeworms *T. solium*, *T. saginata* and *T. asiatica*. It is estimated that 550,000 people are infected nationwide. Echinococcosis caused by *Echinococcus granulosus* and *E. multilocularis* is the most severe parasitic disease in seven western provinces or autonomous regions, including Xinjiang, Ningxia, Gansu, Qinghai, Sichuan, Tibet and Inner Mongolia. Nationwide, there are 380,000 cases of echinococcosis and 66 million people at risk of infection. New cases of echinococcosis have been increasing continuously since 2004 [8]. About 20 million people were infected with trichinellosis caused by *T. spiralis* and *T. nativ*a. With strict measures for the detection and quarantine of contaminated meat and food since the 1990s, the prevention of trichinellosis has become more effective. However, a number of outbreaks of trichinellosis have occurred mainly due to the lifestyle habits of eating raw or undercooked meat, especially in areas inhabited by minor nationalities. Human angiostrongyliasis is caused by the larvae of the rat lungworm *Angiostrongylus cantonensis* and leads to eosinophilic meningitis. Humans become infected by ingesting freshwater and terrestrial snails and slugs. Approximately 400 angiostrongyliasis cases have been reported in China since 1984, mainly from several outbreaks in Zhejiang, Yunnan, Fujian and Beijing.

## Research priorities for parasitic diseases

Recently, emerging food-borne zoonoses, opportunistic parasitic diseases and arthropod borne diseases, and other imported parasitic diseases in China, pose new threats to people’s health. The disease outbreaks and infection rates have increased in the last two decades. In this meeting, several scholars focused their research interests on related diseases and shared their results with the participants. Other scholars presented their results on identifying and monitoring emerging and re-emerging parasitic diseases using advanced technology (see Table 2).

**Table 2:** **Academic reports presented at the 3rd meeting of cross-strait parasitological research, 2013**

*Title*	*Speaker*
The contribution of parasitological research to the history of life science development	Guan-Ling Wu^1^
The transmission control and elimination of the parasitic diseases	Xiao-Nong Zhou^1^
Parasitic diseases and their prevention strategy in Taiwan	Kao-Pin Huang ^2^
Session 1: Food-borne zoonoses	
The screening of pathogenic genes and their functional research in *Clonorchis sinensis*	Xin-Bing Yu^1^
Liver fluke disease epidemic and control situation in Heng county, Guangxi province	Yi-Chao Yang^1^
The investigation on the first outbreak of *Fasciola gigantica* infection in mainland China	Jia-Xu Chen^1^
Sonic hedgehog pathway activation in astrocytes by *Angiostrongylus cantonensis*	Lian-Chen Wang^2^
Perspective of *Taenia asiatica* in Taiwan	Hong-Kean Ooi^2^
Session 2: Opportunistic parasitic diseases	
The genotype, virulence and pathogenesis mechanism of a *Toxoplasma gondii* strain isolated in mainland China	Ji-Long Shen^1^
The existing threat of cryptosporidiosis	Jian-Ping Cao^1^
CRABP2 expression and its related protein-protein interaction network prediction by coincubation of *Blastocystis hominis* and H29 cells *in vitro*	Jyh-Wei Shin^2^
Iron-triggered signal transduction in the protozoan parasite *Trichomonas vaginalis*	Jung-Hsiang Tai^2^
Session 3: Arthropod and vector-borne parasitic diseases	
miRNAome analysis of *Aedes albopictus* and their regulation of dengue virus infection	Xiao-Guang Chen^1^
The research advances in mosquito resistance to insecticides	Chang-Liang Zhu^1^
Multiple pathogens in mosquitoes detected by multiple polymerase chain reaction	Huai-Min Zhu^1^
Tick and Tick-borne diseases	Yi Zhang^1^
The biological research on *Pomacea canaliculata* in mainland China	Shan Lv ^1^
The landscape genetics research on *Oncomelania hupensis*, an intermediate host of *Schistosoma japonicum*	Shi-Zhu Li^1^
Session 4: Translational researches	
The omics research on *Schistosoma japonicum* and its application in innovative diagnosis technology	Wei Hu^1^
Molecular biology technology in the taxonomic identification of the parasites and the application of diagnostic tests	Xin-Quan Zhu^1^
The disease burden of echinococcosis in Tibetan area	Wei-Ping Wu^1^
Re-sequencing of *Trichomonas vaginalis* transcriptomes and genomes	Petrus Tang^2^
Comparative miRNAome in *Trichomonas vaginalis*	Wei-Chen Lin^2^
Diagnosis of Parasitic Infections	K. E. Su^2^
Session 5: Disease monitoring tools	
The imported malaria and schistosomiasis status in mainland China	Ning Xiao^1^
Malaria in Jiangsu province: from control to elimination	Qi Gao^1^
The introduction of veterinary parasitology research in mainland China	Jiao-Jiao Lin^1^
The monitoring, prevention and control of the notifiable infectious diseases including malaria in Taiwan	Dar-Der Ji^2^
Status of *Toxocara canis* infection among schoolchildren in Swaziland and São Tomé and Príncipe of Africa	Ted Chia-Kwung Fan^2^

^1^From mainland China; ^2^From Taiwan.

### Food-borne zoonoses

Emerging and re-emerging food-borne zoonoses have attracted more attention in recent years [22]. The number of outbreaks of food-borne diseases and the number of individuals affected has increased in the last two decades. Human and many animal reservoir hosts (such as dogs, pigs, cats and rats) acquire clonorchiasis via the ingestion of raw fish or shrimps containing infectious metacercariae of *Clonorchis sinensis*. The population with the largest incidence (5.5 million/15 million) resides in the Guangdong province and the second largest in the Guangxi autonomous region. Dr. Yi-Chao Yang reported that an epidemiological survey conducted in the Hengxian county of the Guangxi autonomous region in 2011 indicated that 53.54% of the people were infected with *C. sinensis* because the locals like eating raw fish. Chronic infection with *C. sinensis* can cause liver fibrosis, and the International Agency for Research on Cancer classed it as a Group 1 biological carcinogen in 2009. Dr. Xin-Bing Yu’s team have screened the pathogenic genes from *C. sinensis* to further study their function. Dr. Jia-Xu Chen reported the first outbreak of the *Fasciola gigantica* infection in the Yunnan province, which might be related to the villagers’ eating habits (consumption of raw cordata, coriander, etc.). Additionally, there have been several sporadic food-borne infections recently in Taiwan. Dr. Lian-Chen Wang introduced the history and recent research on *An. Cantonensis*, while Dr. Hong Kean Ooi reviewed the research history and perspective of *T. asiatica* in Taiwan.

### Opportunistic parasitic diseases

Infection rates of opportunistic parasites (such as *Toxoplasma gondii* and *Cryptosporidium* spp.) and sexually transmitted parasites (such as *Trichomonas vaginalis* and pubic louse) have also increased gradually in the last 10 years. Dr. Ji-Long Shen’s team identified the strain of *T. gondii* prevalent in mainland China, and explored its virulence and pathogenesis to the host. Dr. Jian-Ping Cao analysed the current situation of cryptosporidiosis, which is showing a higher infection rate among the HIV-infected population and children. Dr. Jyh-Wei Shin’s team used the microarray and protein-protein interaction prediction techniques to dig out several genes which are highly related to the coincubation between *Blastocystis hominis* and the human colon cell line. In addition, as excess iron can help the growth of *T. vaginalis* and promote its pathogenicity, Dr. Jung-Hsiang Tai’s team observed iron-triggered signal transduction in this protozoan.

### Vector-borne parasitic diseases

Arthropod and vector borne diseases in China remain a serious public health problem. There have been outbreaks of dengue fever transmitted by *Aedes albopictus* in southern China [23]. A similar situation happened in Taiwan. In 2002, the most serious outbreak of dengue fever occurred in Taiwan with 5,285 diagnosed cases. Dr. Xiao-Guang Chen’s team conducted a miRNAome analysis of *Ae. albopictus* and studied the miRNAs’ regulation of the dengue virus infection in mosquitoes. Dr. Huai-Min Zhu utilised multiple polymerase chain reactions for the detection of pathogens in mosquitoes. Dr. Chang-Liang Zhu introduced the research progress on insecticides resistance in mosquitoes after large-scale applications of insecticide. Recently, tick-borne diseases have attracted more attention because of their increasing incidence and consequent significant harm to livestock and human health. Dr. Yi Zhang reported on the distribution of tick-borne diseases in China. Additionally, biological and landscape genetics research on *Pomacea canaliculata* and *O. hupensis*, the intermediate hosts of *An. cantonensis* and *S. japonicum*, respectively, has been carried out by Dr. Shan Lv and Dr. Shi-Zhu Li’s team.

### Translational research

Recent advances in novel technology applications, such as molecular biological, genomic, transcriptomic and proteomic techniques, provide an opportunity to clarify certain parasitologic questions. Regardless of the pathogen’s identification or the basic biological research, these tools are revolutionary for researchers. In this meeting, Dr. Xing-Quan Zhu introduced the progress of applying molecular biological techniques in the identification of various parasites. Dr. Wei-Ping Wu reported on the geographic location data of echinococcosis that were collected by geographical information systems (GIS) in the Tibetan region. In addition, the possibility of whole genome sequencing is being explored in parasitological research. Dr. Wei Hu has integrated parasitology with bioinformatics methods and whole system analyses to provide valuable new insights. Dr. Wei-Chen Lin compared a number of microRNAomes in order to understand the evolution of microRNA expression levels in different conditions. Dr. Petrus Tang’s investigation showed that the genomic organisation and regulation of gene expression by re-sequencing of transcriptomes and genomes is much more complicated than expected. This research leads us to believe that major public health problems will gradually improve with the advancement of technology, and if we can make good use of these new technologies, they will bring impressive scientific achievements.

### Disease monitoring tools

Imported diseases have become the new focus in both mainland China and Taiwan. Over the last 60 years, effective national strategies have successfully brought down the prevalence levels of some important parasitic diseases, such as schistosomiasis japonica and malaria. At present, the critical problem is how to achieve the elimination of these diseases. Therefore, Dr. Xiao-Nong Zhou and Dr. Qi Gao put forward some proposals on how to bring these parasitic diseases from control to elimination in their presentations. Dr. Ning Xiao pointed out in particular that local cases are significantly decreasing, but in contrast, imported cases are increasing annually. Travellers from Africa and foreign workers brought malaria, schistosomiasis mansoni and schistosomiasis haematobium to China, thus active surveillance will be a very important task. In Taiwan, the epidemiology of parasitic infections among foreign workers and foreign brides (including their families and the next generation) need more efforts, as was reported by Dr Kao-Pin Hwang. Dr. Dar-Der Ji introduced the monitoring, prevention and control of the legal infectious diseases, including malaria, in Taiwan. Dr. Chia-Kwung Fan and his team have worked for a long time on toxocariasis in Africa, especially in Swaziland and São Tomé. Based on the results, their research may become a guideline for the zoonosis transmission model.

## Conclusion

In order to take more action relating to the five research priorities aforementioned, the next meeting – the 4th Meeting of Cross-Strait Parasitological Research – will take place at the National Cheng Kung University, in Tainan, in 2015. This meeting will cover all aspects of parasite molecular biology, cell biology, biochemistry, genetics, systems biology, epidemiology and disease control. Based on the warm communications of the previous meetings, we will again try to connect cross-strait parasitologists and encourage more cooperation on scientific research and other aspects.

## Electronic supplementary material

Additional file 1: **Multilingual abstracts in the six official working languages of the United Nations.** (PDF 201 KB)
