# Supplementary material for: Cross-strait parasitological research priorities arrived at by historical tracking and advanced dialogue
Source: Infect Dis Poverty. 2014 Dec 1;3:40. doi: 10.1186/2049-9957-3-40 (PMC4322646; doi:10.1186/2049-9957-3-40)
Supplement: Supplementary file 1 — Additional file 1: Multilingual abstracts in the six official working languages of the United Nations. (PDF 201 KB) [file 40249_2014_84_MOESM1_ESM.pdf]

Translation of the abstract into the six official working languages of the United Nations

التوصل إلى أولويات الأبحاث الطفيلية عبر المضيق عن طريق التتبع التاريخي والحوار المتقدم

جيه وى شين، وجيا شو تشن، ودونغ هوي تشانغ، وى تشن لين، وبو شين، ومين يونيو جي

الملخص: لتعزيز الحوار وتشجيع التعاون عبر المضيق من أجل الوقاية والسيطرة على الأمراض الطفيلية، استعرضت هذه الورقة التقدم والتحديات الراهنة في السيطرة على الالتهابات الطفيلية عبر المضيق والأبحاث التي تمت بهذا الشأن، وفق ثلاثة اجتماعات للأبحاث الطفيلية عبر المضيق في العقد الماضي. وكانت النتيجة الرئيسية للاجتماع الثالث للأبحاث الطفيلية عبر المضيق والذي عقد في أبريل 2013 هي تحديد أولويات الأبحاث الطفيلية.

Translated from English version into Arabic by Wahib Farhan, through

TRANSLATORS
WITHOUT BORDERS

历史沿袭和广泛交流达成海峡两岸寄生虫学优先发展领域的共识

辛致炜、陈家旭、张东辉、林威辰、沈波、季旻珺

摘要：为进一步加强两岸间的学术交流，促进两岸学者在寄生虫病预防和控制等方面的合作，本文简要概述了这十年来举办过三次海峡两岸寄生虫病学术研讨会的历程，总结了目前海峡两岸在寄生虫感染控制和研究中所取得的进展和面临的挑战。在 2013 年 4 月 3 日举行的第三届海峡两岸寄生虫病学术研讨会上确定了今后两岸寄生虫学研究的优先发展领域。

Translated from English version into Chinese by Ji Min-jun

Les priorités en termes de recherche en parasitologie transdétroit ont pu être identifiées grâce à des données historiques et à des discussions approfondies

Jyh-Wei Shin, Jia-Xu Chen, Dong-Hui Zhang, Wei-Chen Lin, Bo Shen, Min-Jun Ji

Description : Afin d'améliorer le dialogue et de promouvoir la coopération transdétroit pour la prévention et le contrôle des maladies parasitaires, ce document passe en revue les progrès et les défis actuels pour le contrôle transdétroit et la recherche des maladies parasitaires, en se basant sur trois réunions transdétroit sur la recherche en parasitologie qui ont eu lieu au cours de ces 10 dernières années. La 3ème réunion sur la recherche en parasitologie transdétroit qui s'est tenue en avril 2013 a eu pour but d'identifier les priorités en termes de recherches en parasitologie.

Translated from English version into French by Fanny, through

Преимущества паразитологического исследования по обе стороны пролива, достигнутые за счет исторического мониторинга и расширенного диалога

Джи-Вей Шин, Джиа-Ксу Чен, Донг-Хуй Джанг, Вей-Чен Лин, Бо Шен, Мин-Джун Джи

Краткий обзор: Чтобы в дальнейшем расширять диалог и способствовать сотрудничеству по обе стороны пролива в целях предупреждения и контроля паразитарных заболеваний, в настоящей работе был рассмотрен прогресс и текущие трудности контроля и исследования паразитарных исследований по обе стороны пролива, исходя из совместных встреч по теме паразитологических исследований, состоявшихся за последнее десятилетие. Основным результатом Третьего совещания по паразитологическим исследованиям, которое прошло в апреле 2013 года, стало выявление научных приоритетов при проведении паразитологического исследования.

Translated from English version into Russian by Irina Zayonchkovskaya, through

Investigaciones parasitológicas relacionadas entre sí y prioridades elaboradas a través del seguimiento de datos históricos y diálogos intensos.

Jyh-Wei Shin, Jia-Xu Chen, Dong-Hui Zhang, Wei-Chen Lin, Bo Shen, Min-Jun Ji

Resumen: Con el objetivo de mejorar el diálogo y promover una estrecha cooperación en la prevención y el control de enfermedades parasitarias, este estudio ha analizado el progreso y los retos actuales en los controles e investigaciones vinculadas entre sí sobre las infecciones parasitarias; se ha basado en tres reuniones sobre investigaciones parasitológicas llevadas a cabo en la última década. El resultado principal de la tercera reunión sobre las investigaciones parasitológicas celebrada en abril de 2013 identificó las prioridades de investigación en parasitología .

Translated from English version into Spanish by Karina Atencio, through
